# A System of Surgery

**Published:** 1847-12

**Authors:** 


ART. II.—*A System of Surgery.* By J. M. CHELIUS, *public Professor of General and Opthalmic Surgery, Director of the Chirurgical and Opthalmic Clinic in the University of Heidelberg, &c., &c.* Translated from the German with Notes and Observations by John F. South, late Professor to the Royal College of Surgeons of England, and one of the Surgeon's to St. Thomas's Hospital; with additional Notes by G. W. Norris of Philadelphia, in three volumes, 8 vo. Published at Philadelphia by Lea and Blanchard, 1847.

Chelius little thought when from the banks of the Rhine he sent out his little "hand-book" upon Surgery, that it would be successively transplanted into England and America, and finally without a thought or a care on his part, grow into three ponderous octavo volumes; and we very much doubt whether, were he to meet his book on these cis-atlantic shores, he would be able to recognize it as his own, so enormously has it swelled and stretched itself beyond its original dimensions.

This work was first published by Chelius at Heidelberg in 1821 as a text book for his students; but the work was so really valuable and was so well received by the profession generally, that in twenty-five years it had passed through six editions in Germany and had been translated into seven different languages. And Mr. South could not have rendered English medical literature a better service than in selecting it for the basis of his labors. Mr. South too was eminently qualified for the duty, both as a translator and annotator. We remember Mr. South well as one of the most talented and indefatigable surgeons of that magnificent charity St. Thomas's Hospital; and we have seen enough of his fugitive contributions to the English Medical Journals to satisfy us fully of his qualifications. The book before us is rather South's Surgery than Chelius's, so completely is the text buried beneath the elaborate but judicious notes and excerpta of Mr. South; the notes added by Mr. Norris being comparatively few. And so long as the fashion is, not to write new books, but only to fill out the margins of old ones, however much we may dislike the fashion, we are compelled to submit; and with what grace we may, we say, this is the best book of notes we have seen.

Since the translation of Heister's great work, more than a century ago, no German author upon Surgery has been translated into our language; and save what we have obtained through the French, English and American authors and journalists second hand, we have known very little of German surgery. But the Germans have not been idle; many of the most valuable improvements and discoveries in surgical operations and surgical pathology have been made by the Germans within the last few years, among which are especially conspicuous the new operations in tenotomy, many improvements in anaplasty, and a vast amount of information upon the surgical diseases of the eye; in which latter department they have fairly outstripped all their cotemporaries. More than fifty large works upon surgery have been published within the present century, whose names have come to our knowledge, and how many more have been issued we have no means of knowing. It is somewhat surprising therefore that for a whole century not one complete German Surgery has appeared in an English dress. We earnestly hope that this may be but the first of a series of translations which shall open to us the whole extent of German improvements in this department of science. Langenbeck, Russ, Walther, Blasius, Meyer, Niemann and many others, are scarcely less deserving the students attention than the work of Chelius; and it will be well for us, while we have bulletined before us constantly the dazzling achievements of the French, whoso restless knives are daily *hitting* upon some new incision, and the steady victories of the English, whose weapons are never drawn save in such straight lines as time and success have sanctioned, to have brought before us the practice of our Saxon cotemporaries, who are neither indebted to chance or custom for their conquests, but who in their studies draw up from the deep fountains of their "inner soul's" those clear and cool thoughts which give to their discoveries the character of inspiration.

Chelius has arranged his subjects under eight divisions, commencing with *Inflammation;* to which division in its various forms and results, he devotes most of the first volume; and for this we think he may find ample apology in the importance and extent of the subject. For whatever we may hold to be the value or necessity of inflammation as a recuperative agent—whether it is or is not a natural and salutary operation of the vital powers, and useful in the restoration of divided parts, it is at least certain, that upon such restoration it is the almost inevitable attendant, and in one form or another, in a greater or less degree of intensity, constitutes the prevailing symptom, and establishes the principal indications of treatment. A knowledge of the phenomena and treatment of inflammation is therefore the corner stone of all surgical practice, which must from the beginning be rightly squared and firmly settled.

"Inflammation" says our author, "is that condition of an organized part in which the vital process and plasticity of the blood are unnaturally raised, and which is manifested by pain, redness, increased temperature, and swelling." This definition of inflammation, like most others, is defective; it fails, we think, entirely to tell us what the thing inflammation is. We certainly find no part of a definition in that portion which informs us how inflammation *manifests* itself, or in other words, what are its symptoms, viz. "pain, redness, heat and swelling"; nor indeed can we be satisfied with that portion of the definition which alone remains, to wit, "inflammation is that condition of an organized part in which the vital process and plasticity of the blood are unnaturally raised." What then distinguishes inflammation from various forms of fever, in which the "vital process and plasticity of the blood are unnaturally raised"? The "vital process" moreover is in health subject to an almost constant efflux and influx, from an infinitude of causes, being always either hastened or retarded by physical or mental action or repose; and even were we able to fix upon and declare what is the exact healthy standard of vital action, we should still be loth to affirm that every upward deviation from this standard constituted inflammation; for then is every "unnaturally raised" pulsation of the heart, or action of the muscles, every "unnaturally raised" functional service of a gland, or of a nerve, inflammation—provided always the other condition be present, viz., increased plasticity of the blood—a condition which exists independently of inflammation—at least independantly of any of the ordinary signs of inflammation—often in the highest degree, in a multitude of diseases; and which condition even in health is subject to very broad variations. Before the *degree* of "vital process" and "plasticity of the blood" can alone determine the definition ot inflammation, their standard of health must be settled, and the amount of elevation necessary to constitute inflammation, and further it must be shown that no other disease presents the same phenomena and has not therefore pre-occupied the ground. This has not yet been done, and the author himself seems to feel the defectiveness of his definition when he adds the following note:

"The elevation of the vital process must be of a certain *duration* and intensity, that is, it must be actually diseased, when we apply to it the name *inflammation*. Thereby alone is inflammation distinguished from the temporary condition of *active congestion* and increased *turgor vitalis*."

But what has the circumstance of *duration* to do with inflammation? Inflammation may be developed in a few seconds, and by reverse many days of increased action, as in fever, may not and does not of itself constitute inflammation, nor in any way distinguish it from *active congestion*. Some *new* circumstance, without reference to duration or intensity, must be superadded, or it remains as at first only increased action. "It must be actually *diseased*," says Chelius; and this is plain enough, but *how* diseased? This is the very point of the inquiry, and io this no answer is given.

The definition furnished by our author has therefore no meaning, yet it is as precise as the definition generally given, and than some it is much better, since it describes correctly certain of the conditions necessary to inflammation, viz., elevation of the vital process and increased plasticity of the blood; and perhaps indeed the definition only lacks that *one* circumttance still unrevealed, which is alone *di gnostic*. We shall not stay to dispute with those who see in inflammation nothing but *diminished* action, and who talk of stimulating the suffering part by bleeding. We agree with Chelius, that in inflammation the "vital process" is raised—that there is unnatural *excitement* of all the tissues actually concerned in the inflammation, whether the current of blood flows more or less rapidly, and whether the vessels are contracted or distended; for with none of these conditions is a state of sur-excitement imcompatable any more than is a state of sur-excitement, or "nervous exciteability," as it is oftener termed, incompatable with extreme nervous debility.

Will the reader now permit us to indulge for a moment in our own speculations upon this much vexed question? Let us assume first as a postulate, what we presume will not be denied. All inflammations are preceeded by *irritation*—irritation of the *nervous* tissue; perhaps originating in the common sensitor nerves, belonging to animal life, as in the case of a wound upon the surface, or originating directly in the ganglionic nerves, as where the irritant is carried into the circulation, and is made directly to impinge upon the nerves distributed upon the walls of the vessels. *Irritation of the nervous tissue* (congestion may produce irritation by mere distention) *is then,* we affirm, *invariably the first link in the chain of circumstances which produces inflammation.* This irritation may be local or general, and its existence is generally indicated by tremors, pain, titillation &c. If continued it produces *fever.*

Fever is a sur-excitement of the nervous system, both animal and organic, but chiefly, we think of the organic system, coupled with sur-excitement of the *vascular* system (including the capillaries,) and its immediate dependencies. This statement will not be so readily admitted, we apprehend, as our definition of irritation, and chiefly because fever presents such a vast variety of forms and characters. But the definition is not designed to include all its phenomena, nor to indicate any of the functional or organic lesions which may result; which latter are so often by modern pathologists made the basis of a definition. We include only those circumstances which we regard as essential to the existence of fever. The excitement of the nervous system is seen in the raised sensibility of the surface, in pains, in restlessness, in tremors, and in the morbid sensibility of the mind. If sooner or later the sensibility becomes unnaturally obtunded, it is as a consequence. The excitement of the vascular system is indicated by increased frequency and force of the pulse, by redness of the surface, &c. If the contrary phenomena occur, it is again only as a "consequence" of previous excitement. While the "immediate dependencies" of the vascular system—the absorbents, secernents, &c., declare their participation by spasmodic and firm occlusion of their mouths. Secretions are diminished or entirely arrested in the bronchial passages, in the intestinal canal, in the kidneys, upon the skin and wherever else secernents may be found. While absorption through the skin is rendered difficult, the lacteals refuse to act and other similar phenomena indicate excitement and spasm at their orifices.

We assume farther, that fever, like its precedent irritation, may be local as well as general—a blush is a local fever—the first redness produced by a blister or by any irritant is the same, and in a limited space presents all the phenomena of general fever—but in order that the fever may become permanent it is necessary that the irritant should be excessive or persistent.

We are now prepared to offer our definition of inflammation. That inflammation may be developed in any organized vital structure it is only necessary that the irritation should be pushed beyond the point required for the production of fever: when, the secernents still remaining closed, so that no relief to the distended vessels can be obtained through them, an *effusion* occurs, or a transfusion of some of the contents of the vessel through its parietes. The matter effused may be blood, serum, lymph, &c. It is this new condition, effusion, which, superadded to the irritation and fever constitutes the only pathologic distinction between fever and inflammation. We cannot be misunderstood. We do not regard effusion as in itself constituting inflammation, but we affirm that effusion with the other circumstances mentioned, is the thing which we and others call inflammation. The definition which we propose to substitute, for our authors is then—*inflammation is a sur-excitement of the nervous and vascular systems, with the immediate dependencies of the latter, accompanied with effusion.*

"Inflammation always commences with a more or less intense pain; the sensibility of the part is increased, redness soon follows, and blood appears in vessels where previously it had not been observed; the temperature of the part is raised, its functions disturbed, secretion suppressed, (at least at first,) or changed, perspiration diminished, and the part swelled."—Or in the language of the old writers, "*rubor, tumor, calor cum dolore*" are the signs of inflammation. But our author must certainly know that not one of these signs are constant, and therefore not one can be considered as diagnostic. *Pain* is common to irritation, fever and inflammation, and by reverse it may be absent in either—thus occasionally in the most destructive inflammations of joints, the lungs, liver, kidneys, &c., no pain is present. *Redness* and *heat* are also common to both fever and inflammation, but redness does not generally exist when tendons, ligaments, &c., are inflamed. Some writers, also, have chosen to consider *heat* as "the only unequivocal sign of inflammation," but with what right we are not able to understand. *Swelling* too, occurs in oedema, and alone it cannot indicate inflammation. What shall we say then—are there no positive signs of inflammation? We affirm that there are. As "effusion" when accompanied with irritation and fever constitutes the pathological condition of inflammation, so, also, "swelling" (which results inevitably from "effusion") when accompanied with a sur-excitement of the same systems must certainly constitute the pathognomonic sign. A swelling always exists in inflammation—not always manifest, because the tissue inflamed may be within; and in some cases when it is more external, as in the inflammations of fibrous structures or bones, the firm and unyealding character of the textures will not admit of any sensible enlargement in their periphery; yet it is none the less true that an effusion has taken place into their cellules, and that the part inflamed, i. e., the intimate cellular structure of the part, is engorged and swollen. Since, however the swelling is not always manifest, it cannot be to us an invariable test of inflammation; but what we affirm is, that *when it is* manifest, the other conditions being present, it is a certain sign of inflammation. The same cannot be said justly of either of the other signs mentioned.

From Celsus to Chelius all writers have enumerated these four local signs and no more. But as we have presumed to give our own doctrines of inflammation without regard to "established opinions," we shall not now hesitate to declare a "new sign" even at the risk of being regarded sinfully heterodox.

*Tenderness*, is the "new sign," which we preach—not new to the observing practitioner, nor indeed to the world—but new only to the books. If the patient complains to-day of pain in the knee joint, we say it may be a nervous pain, and only sympathetic with disease in the hip-joint. It may be irritation, and not inflammation. But if to-morrow it is swollen, and hot, it is another thing; we now pronounce it certainly a case of inflammation of this joint. Here, because the "swelling" is manifest we need no other evidence of inflammation. Again, if the patient complains to-day of pain in his loins, in the region of the kidneys, we may also pronounce it nervous, mere irritation, direct or sympathetic; but to morrow although it is neither red, nor increased in temperature nor swollen indeed, and yet if on pressure we discover a *tenderness*, "Ah," we say, "here is inflammation." We distinguish peritonitis from colic, not by the pain, nor perhaps by the heat, or tumerfaction, but chiefly, and in some instances solely, by the tenderness. Tenderness is the symptom which, if not infallible, is at least the *most* infallible of any heretofore given; and we appeal to our readers whether the complete absence of this circumstance would not in all cases, where pressure could be made directly upon the diseased part, forbid us from regarding it as inflamed.

It is scarcely essential to this matter whether we can show, in what consists the pathological distinction between pain and tenderness, or not, since there is really a wide practical difference. Yet we will offer what seems to us a probable explanation.

Pain may be produced by the point of a pin; and equally upon a surface which is so diseased as to be capable of developing the sensation of tenderness, as upon a healthy surface. If we prick an inflamed structure it produces pain only, precisely as when we prick a healthy structure. It is, we think, a single sensation, and is the result of the disturbance of a single nervous filament, or bundle of filaments: while tenderness is a complex sensation, and is the result of the simultaneous disturbance of a large number of separate filaments; it cannot be produced by the point of a pin, but it is readily developed by pressure with a broader surface, as the end of the finger. It exists in inflammation, as a consequence of effusion: the matter effused being crowded into confined spaces or loculi, whose walls are supplied with an infinite number of terminal nerves, upon which unwonted pressure is made; and it now requires only an opposing broad surface to still more compress these filaments, until the sensation of tenderness shall result.

"The *results* of inflammation are *resolution*, *exudation*, *suppuration ulceration*, *induration*, *and various other transformations of organs*, *softening* and *mortification*. All these conditions, excepting resolution, are merely different living processes, which are brought about by inflammation, and are still accompanied by it for a long time."

Chelius has left the beaten track which nearly all writers have taken before him, and has spoken, intelligibly of "results," rather than of "terminations." And the student is not left to understand that when these circumstances occur, the inflammation is terminated. Some are concomitants, some consequences, and some terminations. Exudation or effusion, suppuration, ulceration, induration, and to these we may add the granulation, adhesion and cicatrization, of other writers, we regard as indifferently either concomitants or consequences, yet they are all results. Resolution, and death, or mortification, alone are terminations.

On the subject of that peculiar affection of children which we have been accustomed to term "cancrum oris," upon which an excellent article may be found in the second volume of the North American Medical and Surgical Journal from the pen of B. H. Coates, and also another by Dr. S. Jackson in the 12th volume of the Medical Recorder, (called by Jackson *gangroenopsis*). Our author remarks:—

"Here must also be mentioned that mortification of the cheek which has been called *Noma* by Vogel. It is fortunately not frequent, as it is a horrible and generally fatal disease. With a single exception, of the half dozen cases I have seen, all were children under four or three ears old; some idiopathic, and others originating in a sloughing of the mucous membrane of the mouth, under the careless use of mercurials; and though generally in unhealthly subjects, yet the disease also occured in robust, well-fed children. In its idiopathic form it has been well described, by Drs. Evanson and Maunsell, as follows:—"A particular form of gangrene of the mouth without any preceding inflammation occasionally attacks infants, especially such as are feeble at birth or broken down by disease. An œdematous circumscribed swelling appears on the cheek, with a central point, more or less hard, over which occurs a dark-red spot. This spot may appear on the inside or outside of the cheek; and the skin over the œdematous part is character ized by an oily appearance. An eschar forms from within outwards on the central point, and the soft parts mortify, often extensively, down to the bone, so that the parietes of the cheeks and gums are destroyed, falling off in shreds, mixed with a dark sanguineous fluid, and accompanied by a very fetid odour." In neither of my cases, excepting the adult, did I witness the begining of the disease; but gangrene to a greater or less extent of one cheek, involving generally the corresponding half of the upper lip, existed when the children were brought to me; the surrounding parts were tumid, hard, and of a dull yellow-white hue, very similar to the characteristic color of the countenance of patients under malignant disease. I have little doubt that the mortification of the mouth and fauces after measles, mentioned by Huxham, as well as those referred to by Marshall Hall, and by him stated to have happened after previous disorder of the digestive organs, typhus fever, or some inflammatory disease, are of precisely the same character as those resulting from mercurial influence. The little patient, if not already in a typhoid state, soon falls into it, rapidly sinks as the gangrene spreads, and quickly dies; often, indeed before the least attempt at separation of the slough has been made. Usually three or four days are sufficient to destroy life, but, in one instance, I recollect a child of two years old having lived for a fortnignt, and the greater part of the gangrenous cheek had separated, leaving one side of the cavity of the mouth completely exposed. I fully agree with Evanson and Maunsell, that "no disease can be more frightful or formidable than sloughing of the mouth in children. Recovery seems impossible, when once the disease has set severely in, the child sinking beneath the constitutional disturbance, independent of the local ravages of the disorder, which, however, are often such as to render recovery not to be desired, so frightful is the deformity necessarily entailed."

Following the various forms of mortification, Chelius treats next of some peculiar kinds of inflammation such as, erysipelas, burns, frost-bite, boils, and carbuncles; then of inflammations of special organs, commencing with the tonsils. In all of these chapters we find much original and interesting matter, but we have speculated so long upon the subject of inflammation that we are compelled to ask our readers to accept our opinion of the worth of this volume, or, as we honestly believe they will find to their profit, they must read it for themselves. In our notice of the second and third volumes we will endeavor to keep somewhat nearer the text.

